# Association of metabolites on ischemic stroke subtypes: a 2-sample Mendelian randomization study

**DOI:** 10.3389/fneur.2024.1417357

**Published:** 2024-08-29

**Authors:** Jingyuan Zhang, Anning Wang, Yanyan Zhao, Luping Ma, Hui Shen, Weikai Zhu

**Affiliations:** ^1^First Affiliated Hospital of Dalian Medical University, Dalian, China; ^2^Institute (College) of Integrative Medicine, Dalian Medical University, Dalian, China; ^3^Dalian Dermatosis Hospital, Dalian, China

**Keywords:** metabolites, ischemic stroke subtypes, Mendelian randomization study, large artery stroke, cardioembolic stroke, small vessel stroke

## Abstract

**Background:**

Metabolomics is increasingly being utilized in IS research to elucidate the intricate metabolic alterations that occur during ischemic stroke (IS). However, establishing causality in these associations remains unclear between metabolites and IS subtypes. In this study, we employ Mendelian randomization (MR) to identify specific metabolites and investigate potential causal relationships between metabolites and IS subtypes.

**Methods:**

MR analysis was conducted using genome-wide association study (GWAS) summary data. We obtained 1,091 blood metabolites and 309 metabolite ratios from the GWAS Catalog (GCST90199621-90201020), which gene sequencing data from 8,299 individuals from the Canadian Longitudinal Study. We obtained GWAS summary statistics for IS subtypes which include large artery stroke (LAS), cardioembolic stroke (CES), and small vessel stroke (SVS) from the MEGASTROKE consortium that included 446,696 cases of European ancestry and 406,111 controls of European ancestry. The primary analysis utilized inverse-variance weighted (IVW) method. To validate our results, we performed supplementary analyses employing the MR-Egger, weighted median, simple mode, and weighted mode methods. Heterogeneity and pleiotropy were assessed through Cochran’s *Q* test, MR-Egger intercept test, and leave-one-out analysis.

**Results:**

The study assessed the possible causality of serum metabolites in the risk of IS subtypes. The discovery of significant causal links between 33 metabolites and 3 distinct IS subtypes.

**Conclusion:**

Metabolites show significant potential as circulating metabolic biomarkers and offer promise for clinical applications in the prevention and screening of IS subtypes. These discoveries notably advance our comprehension of the molecular processes specific to IS subtypes and create avenues for investigating targeted treatment approaches in the future.

## 1 Introduction

Stroke represents a highly prevalent neurological disorder and constitutes a principal cause of disability and mortality among middle-aged and elderly populations (1). It poses a significant public health challenge worldwide. There are 6.3 million deaths caused by stroke (2). Ischemic stroke (IS) is frequently encountered in stroke and can be divided into large artery stroke (LAS), cardioembolic stroke (CES), and small vessel stroke (SVS) (3). Diagnosis and classification of IS are predominantly determined by risk factor profiles, stroke’s clinical manifestations, and results from brain imaging studies, including CT or MRI (3). Multiple studies have illustrated that various subtypes of IS entail the demise of nerve cells (4). However, the biological processes and risk factors underlying the incidence of ischemic stroke remain elusive, notwithstanding extensive research efforts.

Metabolomics is increasingly being utilized in IS research to elucidate the intricate metabolic alterations that occur during IS (4–6). Metabolites serve as a diagnostic biomarker, enabling the prediction of stroke outcomes (6). This approach not only illuminates the underlying mechanisms of IS but also facilitates the development of personalized treatment strategies (7, 8). However, our current understanding of the metabolic profile in IS patients across different subtypes remains limited.

Mendelian randomization (MR) is a genetic epidemiological statistical method used for causal inference in cross-sectional research. It utilizes genetic variations related to the exposure factor of interest as instrumental variables to estimate the causal effect of the exposure factor on disease outcomes or other variables in cross-sectional study data (9). One of the key advantages of the MR is that genetic variants are randomly and independently assigned to the population, making them stable throughout a person’s life. This allows the MR method to effectively address the influence of confounding factors and achieve causal inference (10).

In the study, we used GWAS summary data to conduct a two-sample MR study. The objective was to identify specific metabolites and investigate potential causal relationships between metabolites and IS subtypes.

## 2 Materials and methods

### 2.1 Data sources on the serum metabolites

We obtained 1,091 blood metabolites and 309 metabolite ratios from the GWAS Catalog (GCST90199621-90201020), which gene sequencing data from 8,299 individuals from the Canadian Longitudinal Study (11).

### 2.2 Instrumental variables selection

To investigate the causal effect of blood metabolites and metabolite ratios on IS across different subtypes, we obtain instrumental variables selection **(**IVs). Firstly, we selected SNPs with a correlation *p* < 1 × 10^−5^. Additionally, we applied a linkage disequilibrium (LD) threshold of *R*^2^ < 0.001 and a clumping distance of 10,000 kb by using “TwoSampleMR” packages. These stringent criteria ensured that the selected SNPs were independent and not in strong linkage disequilibrium with each other. By utilizing thresholds, we aimed to increase the number of eligible SNPs available for sensitivity analysis and to maximize the proportion of genetic variation that the genetic predictors could explain. After extracting the relevant information for each SNP, we calculated the proportion of interpreted variation (*R*^2^) and *F* statistics to quantify the strength of the instrumental variable. The *F* statistic is commonly employed to assess the effectiveness of instruments and is calculated using the formula *F* = *R*^2^ × (*N* − *k* − 1)/*k* (1 − *R*^2^), where *R*^2^ represents the proportion of variance explained by the instruments. It is calculated using the formula *R*^2^ = 2 × MAF × (1 − MAF) × *β*^2^, *N* represents the sample size and *k* denotes the number of selected IVs. In this study, we set a standard cutoff value of *F* statistic >10 to mitigate the potential for weak instrument bias (Supplementary material S1).

### 2.3 Data sources on the IS subtypes

We obtained GWAS summary statistics for IS subtypes which include LAS, CES and SVS from the MEGASTROKE consortium that included 446,696 cases of European ancestry and 406,111 controls of European ancestry (12).

### 2.4 Statistical methods

This study is reported following the Strengthening the Reporting of Observational Studies in Epidemiology Using Mendelian Randomization guidelines (STROBE-MR, Supplementary Table S2). We employed different MR methods to assess the possible causal link between blood metabolites, metabolite ratios, and IS subtypes. We will further validate the results using four more MR approaches if the inverse variance weighted (IVW) method establishes a significant causal association (*p* < 0.01): Weighted median, basic mode, weighted mode, and MR-Egger (13). These supplementary MR methods improve the consistency and robustness of our results. For identifying and addressing any biases brought about by pleiotropy, the MR-Egger approach is especially helpful. The weighted median approach provides a more reliable estimate when more than 50% of the IVs are invalid (14). The estimates derived from various instrumental variables can be combined by employing either the simple or weighted mode methods as alternatives. By employing these multiple MR methods, we aim to obtain a comprehensive understanding of the potential causal connection between blood metabolites, metabolite ratios, and IS subtypes. Finally, odds ratios (OR) and 95% confidence intervals (CI) were used to present the results of causal connections.

**Figure 1:**
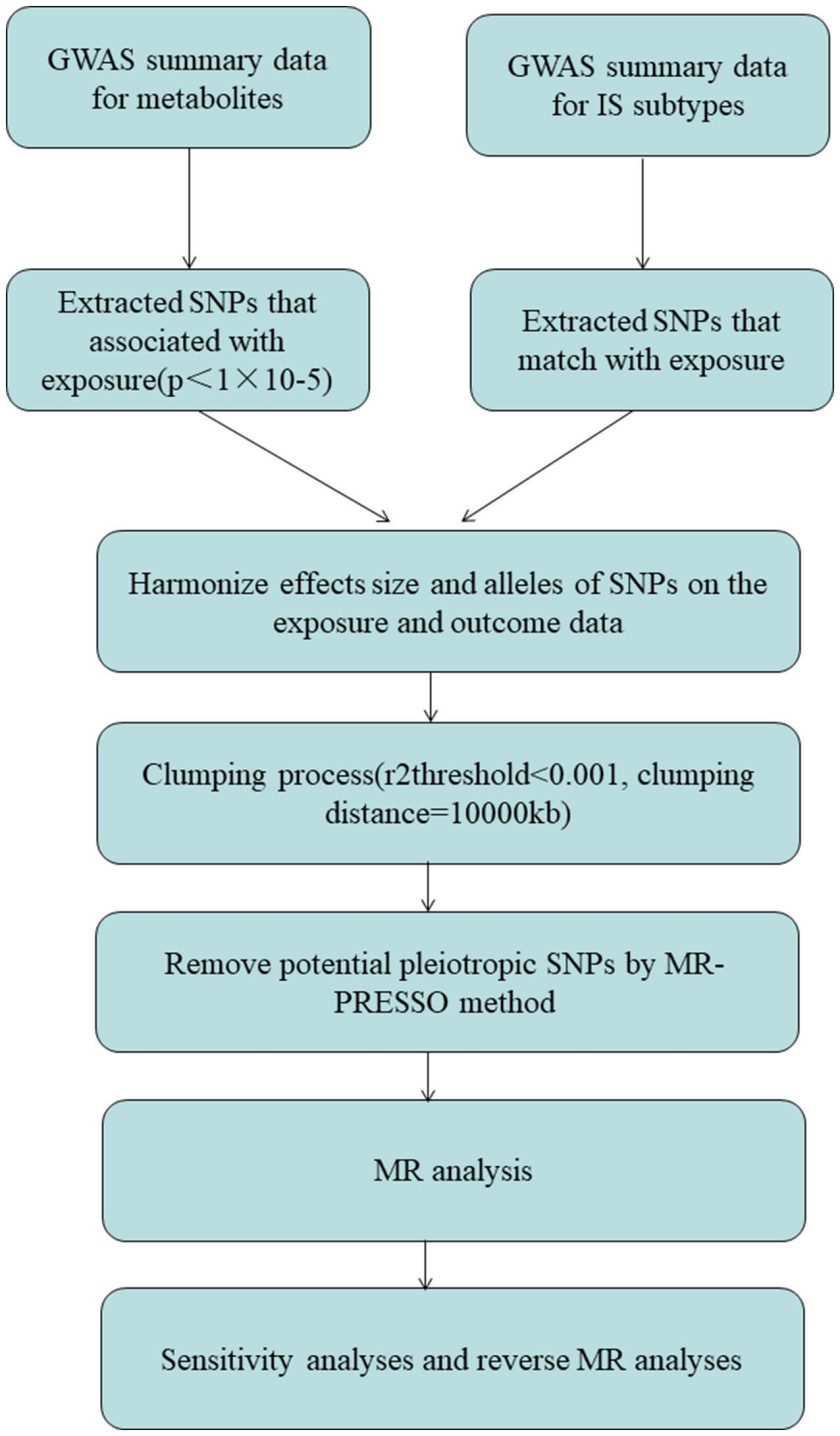
The diagram of MR analysis processing. SNPs, single nucleotide polymorphisms; GWAS, genome-wide association study; MR, Mendelian randomization; MR-PRESSO, Mendelian Randomization Pleiotropy RESidual Sum and Outlier (MR-PRESSO) test.

Heterogeneity across estimates of genetic instruments may be assessed using funnel plots and Cochran’s *Q* statistic, with a significant *p*-value threshold of 0.05 (10). Furthermore, we utilized the MR-Egger intercept test, employing a significant *p*-value cutoff of 0.05, to detect the presence of horizontal pleiotropy (15). The results are visually presented using scatter plots (16). To test the reliability of our conclusions, we implemented leave-one-out analyses, repeatedly performing the IVW analysis while excluding one exposure-related SNP at a time. This iterative approach enabled us to assess the robustness of our results by examining the individual SNP contributions to the causal connection between blood metabolites, metabolite ratios, and IS subtypes. By employing these combined methodologies, we aimed to ensure the validity and reliability of our findings (14, 17, 18). Furthermore, to strengthen the reliability of our MR studies, we conducted replicated analyses by removing relevant confounders from IVs. Specifically, we obtained the confounder-related SNPs from the PhenoScanner V2 database. This step allowed us to address potential confounding factors and enhance the reliability of our MR analyses (Figure 1).

The analyses in this study were conducted using R software (version 4.3.1). For our MR investigation, we utilized two R packages: “TwoSampleMR” and “MRPRESSO.”

## 3 Results

### 3.1 MR analysis

**Figure 2:**
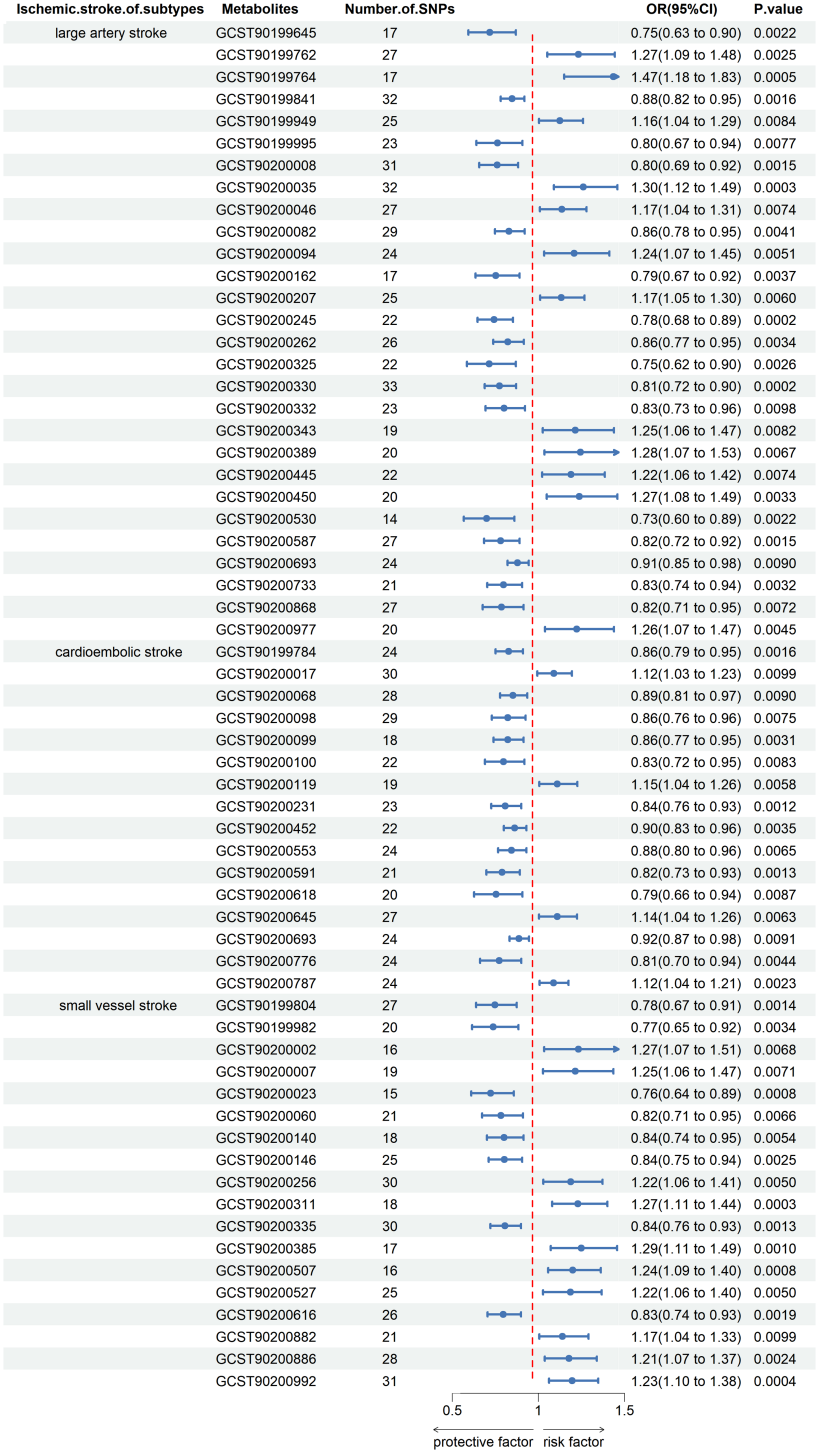
Forest plot shows the expression causality of metabolites for ischemic stroke subtypes.

We performed MR analysis to assess the causal association of 1,091 blood metabolites and 309 metabolite ratios with IS. The results assessed by the IVW-FE showed that N6-carbamoylthreonyladenosine levels (GCST90199762), N2,N2-dimethylguanosine levels (GCST90199764), 6-oxopiperidine-2-carboxylate levels (GCST90199949), palmitoyl dihydrosphingomyelin (d18:0/16:0) levels (GCST90200035), 1-stearoyl-2-docosahexaenoyl-gpc (18:0/22:6) levels (GCST90200046), ascorbic acid 2-sulfate levels (GCST90200094), methyl vanillate sulfate levels (GCST90200207), N-formylmethionine levels (GCST90200343), leucine levels (GCST90200389), caprylate (8:0) levels (GCST90200445), caproate (6:0) levels (GCST90200450), arachidonate (20:4n6) to paraxanthine ratio (GCST90200977) were associated with an increased risk for LAS, while quinate levels (GCST90199645), glycocholenate sulfate levels (GCST90199841), sphingomyelin (d18:1/20:2, d18:2/20:1, d16:1/22:2) levels (GCST90199995), C-glycosyltryptophan levels (GCST90200008), 1-oleoyl-2-linoleoyl-GPE (18:1/18:2) levels (GCST90200082), 3-hydroxyphenylacetoylglutamine levels (GCST90200162), eicosenedioate (C20:1-DC) levels (GCST90200245), N-succinyl-phenylalanine levels (GCST90200262), Glycerol levels (GCST90200325), 1-palmitoyl-2-linoleoyl-gpc (16:0/18:2) levels (GCST90200330), 1-palmitoyl-2-linoleoyl-GPI (16:0/18:2) levels (GCST90200332), X-17335 levels (GCST90200530), X-21733 levels (GCST90200587), glycochenodeoxycholate glucuronide (1) levels (GCST90200693), adenosine 5′-diphosphate (ADP) to N-acetylglucosamine to N-acetylgalactosamine ratio (GCST90200733), isoleucine to phosphate ratio (GCST90200868) were associated with a decreased risk for LAS. Sphingomyelin (d17:1/16:0, d18:1/15:0, d16:1/17:0) levels (GCST90200017), glycosyl ceramide (d18:1/23:1, d17:1/24:1) levels (GCST90200119), X-24565 levels (GCST90200645), glutamine to asparagine ratio (GCST90200787), were associated with an increased risk for CS, while cysteine-glutathione disulfide levels (GCST90199784), 1,2-dilinoleoyl-GPE (18:2/18:2) levels (GCST90200068), ceramide (d18:1/24:1) levels (GCST90200098), N-oleoylserine levels (GCST90200099), perfluorooctanesulfonate (PFOS) levels (GCST90200100), lithocholate sulfate (1) levels (GCST90200231), plasma free asparagine levels (GCST90200452), X-17653 levels (GCST90200553), X-21383 levels (GCST90200591), X-23782 levels (GCST90200618), lycochenodeoxycholate glucuronide (1) levels (GCST90200693), glutamate to glutamine ratio (GCST90200776) were associated with a decreased risk for CS. 6-hydroxyindole sulfate levels (GCST90200002), 3-methoxycatechol sulfate (2) levels (GCST90200007), dibutyl sulfosuccinate levels (GCST90200256), 4-acetamidobutanoate levels (GCST90200311), sphingosine levels (GCST90200385), X-12822 levels (GCST90200507), X-13866 levels (GCST90200527), aspartate to mannose ratio (GCST90200882), phenylpyruvate to 4-hydroxyphenylpyruvate ratio (GCST90200886), alanine to asparagine ratio (GCST90200992) were associated with an increased risk for SVS, while 7-alpha-hydroxy-3-oxo-4-cholestenoate (7-hoca) levels (GCST90199804), 4-vinylguaiacol sulfate levels (GCST90199982), trans 3,4-methyleneheptanoate levels (GCST90200023), 1-(1-enyl-palmitoyl)-2-linoleoyl-GPC (p-16:0/18:2) levels (GCST90200060), carotene diol (2) levels (GCST90200140), docosahexaenoylcarnitine (C22:6) levels (GCST90200146), stearoyl sphingomyelin (d18:1/18:0) levels (GCST90200335), X-23659 levels (GCST90200616), were associated with a decreased risk for SVS (Figure 2).

In addition, four additional methods, MR-Egger, weighted median, simple mode, and weighted mode, were performed to assess the causal effect of these 1,091 blood metabolites and 309 on LAS, CES, and SVS (Supplementary material S4).

### 3.2 Sensitive analysis

**Figure 3:**
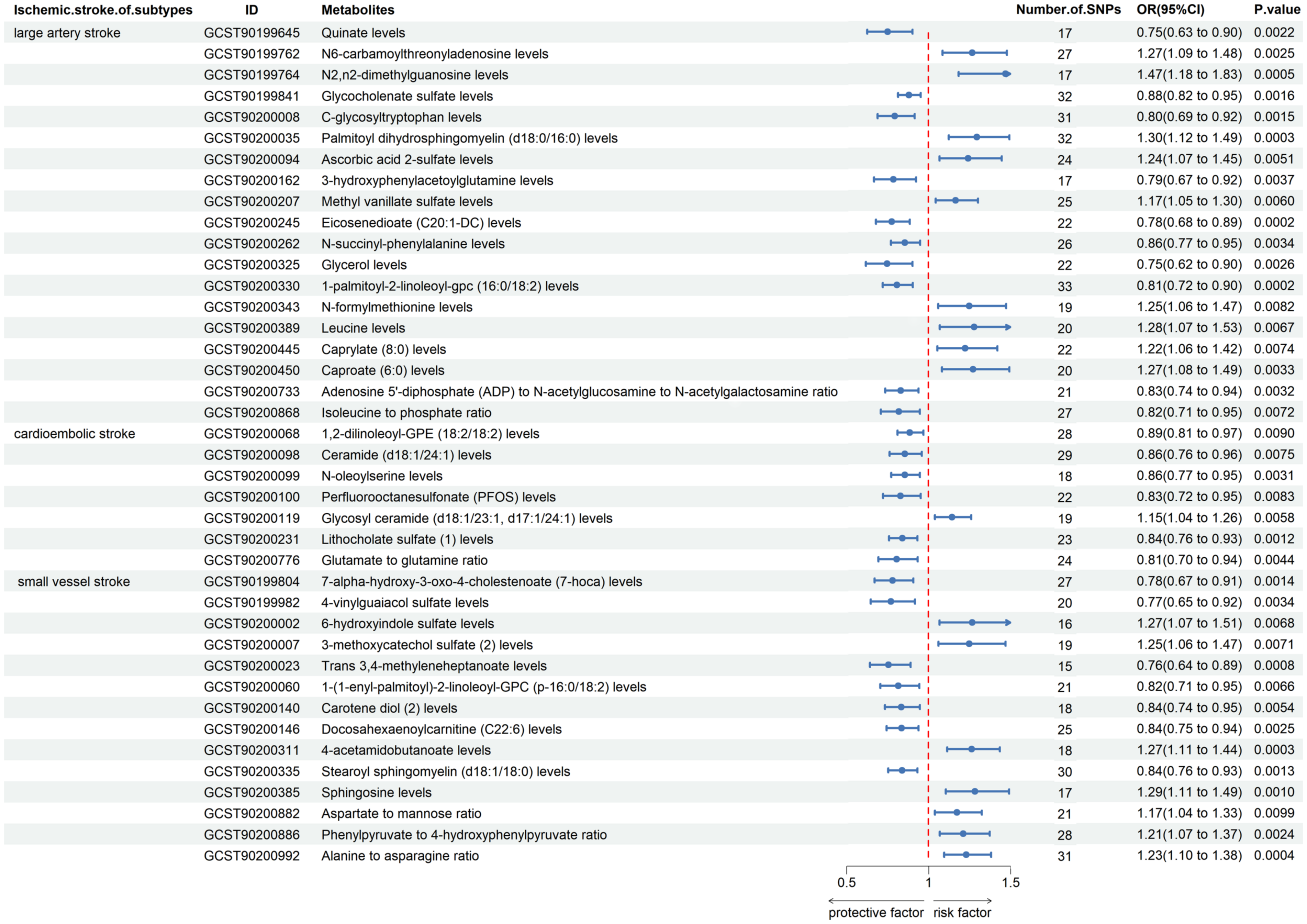
Forest plot shows the expression causality of metabolites for ischemic stroke subtypes following sensitivity analysis.

Firstly, the results of Cochran’s *Q* test indicated the absence of heterogeneity. Subsequently, eicosenedioate (C20:1-DC) levels, ceramide (d18:1/24:1) levels, dibutyl sulfosuccinate levels, 3-methoxycatechol sulfate (2) levels showed horizontal pleiotropy by using the MR-Egger intercept test (*p* > 0.05) and MR-PRESSO global test (*p* > 0.05) (Supplementary materials S2, S3). Finally, the leave-one-out analysis (*p* > 0.05) proved the unreliability of the MR data in 6-oxopiperidine-2-carboxylate levels, sphingomyelin (d18:1/20:2, d18:2/20:1, d16:1/22:2) levels, 1-stearoyl-2-docosahexaenoyl-gpc (18:0/22:6) levels, 1-oleoyl-2-linoleoyl-GPE (18:1/18:2) levels, 1-palmitoyl-2-linoleoyl-GPI (16:0/18:2) levels, arachidonate (20:4n6) to paraxanthine ratio, cysteine-glutathione disulfide levels, sphingomyelin (d17:1/16:0, d18:1/15:0, d16:1/17:0) levels, plasma free asparagine levels, glycochenodeoxycholate glucuronide (1) levels, glutamine to asparagine ratio since excluding some IV did shift the results. Reorganization of the forest plot after the presentation of these metabolites (Figure 3).

### 3.3 Replicated analysis after removing confounders-related IVs

In the study, we identified 40 blood metabolites and metabolite ratios that were associated with IS subtypes. However, some of these blood metabolites and metabolite ratios were also found to be associated with other factors such as body mass index, high blood pressure, self-reported hypertension, self-reported atrial fibrillation, venous thrombosis (Table 1; Supplementary material S5). To investigate the causal associations of these blood metabolites and metabolite ratios with IS subtypes, the researchers removed the SNPs that were associated with these confounding factors from the IVs and re-evaluated the causal associations using MR analysis and sensitive analysis.

**Table 1:** Details of the genetic variants with potential pleiotropy among instrumental variables used for blood metabolites and metabolite ratios.

SNP	Trait	*p*-value
rs1047891	Body mass index	2.65 × 10^−6^
rs1047891	Vascular or heart problems diagnosed by doctor: high blood pressure	9.30 × 10^−8^
rs1047891	Self-reported hypertension	2.37 × 10^−7^
rs10993370	Self-reported atrial fibrillation	3.90 × 10^−8^
rs11244061	Venous thrombosis	3.20 × 10^−14^
rs11244061	Phlebitis and thrombophlebitis	1.81 × 10^−20^
rs11693959	Alcohol intake frequency	6.21 × 10^−7^
rs12316258	Atrial fibrillation and flutter	4.60 × 10^−8^
rs1260326	Alcohol intake frequency	7.60 × 10^−40^
rs1260326	Alcohol consumption	1.00 × 10^−21^
rs1260326	Diabetes diagnosed by doctor	7.62 × 10^−13^
rs12607820	Alcohol intake frequency	1.16 × 10^−6^
rs141471965	Body mass index	2.31 × 10^−6^
rs1728911	Alcohol intake frequency	6.83 × 10^−14^
rs2021965	Body mass index	2.11 × 10^−6^
rs2325676	Alcohol usually taken with meals	7.09 × 10^−6^
rs270607	Vascular or heart problems diagnosed by doctor: high blood pressure	6.92 × 10^−6^
rs34671296	Body mass index	6.75 × 10^−6^
rs445925	Presence of carotid artery plaque	4.00 × 10^−6^
rs445925	Atherosclerosis	2.00 × 10^−8^
rs445925	Atherosclerosis	4.00 × 10^−6^
rs445925	Carotid intima media thickness	4.00 × 10^−6^
rs445925	Carotid intima media thickness	2.00 × 10^−8^
rs445925	Common carotid artery intima media thickness IMT	1.70 × 10^−8^
rs4500751	Body mass index	8.90 × 10^−6^
rs45499402	Body mass index	2.39 × 10^−6^
rs4665972	Alcohol intake frequency	1.29 × 10^−36^
rs4665972	Diabetes diagnosed by doctor	4.43 × 10^−12^
rs4997081	Vascular or heart problems diagnosed by doctor: high blood pressure	8.59 × 10^−18^
rs4997081	Self-reported hypertension	1.79 × 10^−17^
rs56113850	Number of cigarettes currently smoked daily	7.33 × 10^−19^
rs56113850	Smoking status: current	5.27 × 10^−15^
rs56113850	Smoking status: previous	3.69 × 10^−8^
rs62132803	Alcohol intake frequency	1.10 × 10^−6^
rs67402452	Body mass index	6.81 × 10^−6^
rs67402452	Atrial fibrillation and flutter	4.94 × 10^−23^
rs67402452	Self-reported atrial fibrillation	2.32 × 10^−15^
rs7203642	Vascular or heart problems diagnosed by doctor: high blood pressure	1.13 × 10^−17^
rs7203642	Self-reported hypertension	2.38 × 10^−17^
rs77924615	Vascular or heart problems diagnosed by doctor: high blood pressure	1.13 × 10^−17^
rs77924615	Self-reported hypertension	3.32 × 10^−17^
rs780093	Alcohol intake frequency	1.21 × 10^−39^
rs780093	Diabetes diagnosed by doctor	2.87 × 10^−12^
rs8113105	Atrial fibrillation and flutter	3.90 × 10^−6^
rs9928003	Self-reported hypertension	6.84 × 10^−18^

**Figure 4:**
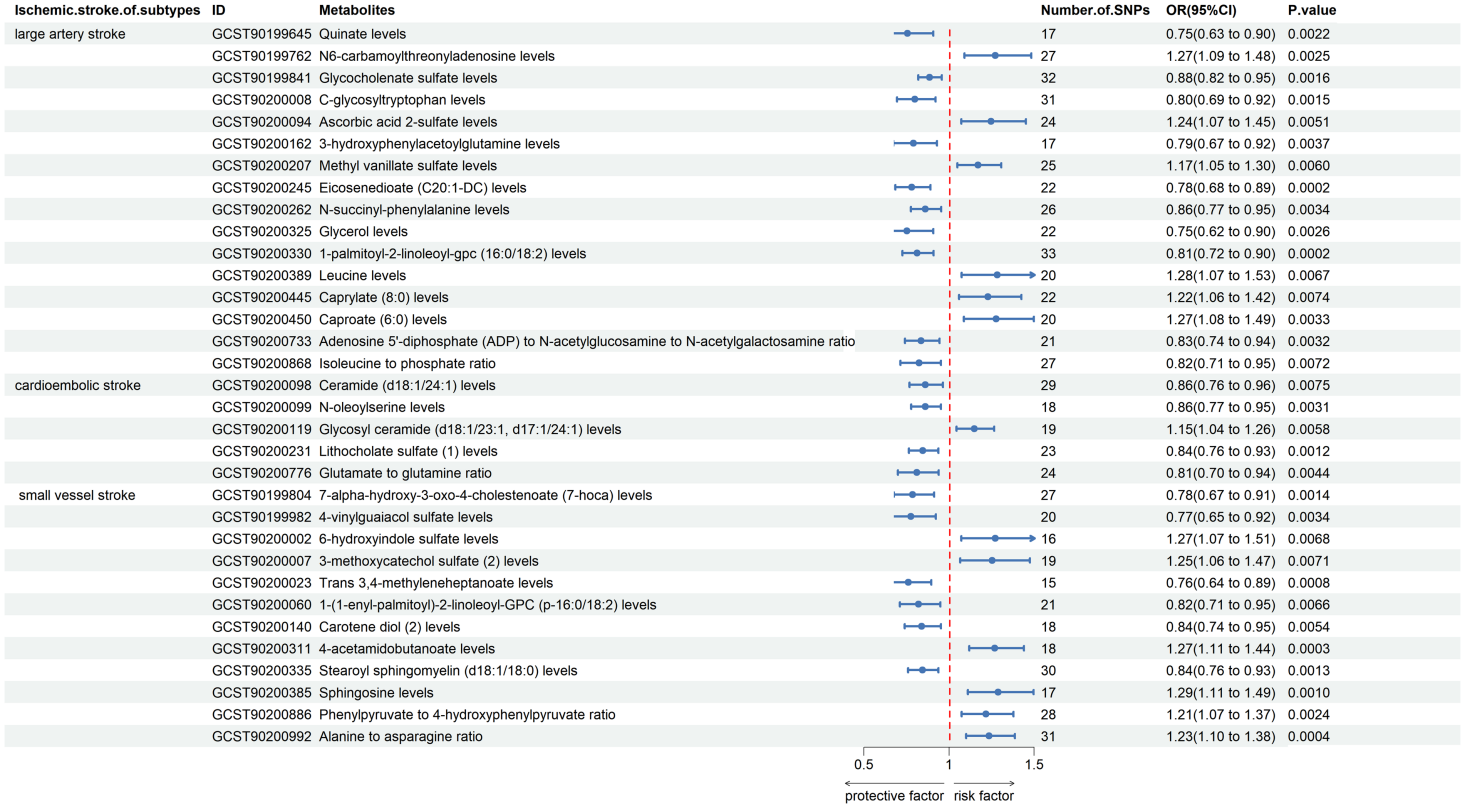
Forest plot shows the expression causality of metabolites for ischemic stroke subtypes following replicated analysis subsequent to the removal of confounder-related IVs.

The results indicated that the causal effects of blood metabolites and metabolite ratios, with the exception of N2,N2-dimethylguanosine levels, Palmitoyl dihydrosphingomyelin (d18:0/16:0) levels, N-formylmethionine levels, 1,2-dilinoleoyl-GPE (18:2/18:2) levels, perfluorooctanesulfonate (PFOS) levels, docosahexaenoylcarnitine (C22:6) levels, aspartate to mannose ratio, remained significant (Figure 4; Supplementary material S6). This approach helps to isolate the effects of blood metabolites and metabolite ratios on IS subtypes by accounting for the potential influences of these confounding factors.

## 4 Discussion

The past few decades have witnessed remarkable advances in metabolomics as a valuable tool for accurately identifying disease biomarkers, enabling a deeper understanding of the disease processes underlying strokes (4, 19, 20). Most studies were animal or case-control studies, which can demonstrate an association with stroke but cannot establish a causal relationship. In this MR study, we identified 17 blood metabolites and metabolite ratios in LAS, 5 in CS, and 12 in SVS that may serve as distinct metabolomic signatures associated with different IS subtypes, potentially aiding in etiology and prognosis determination.

The results of the study suggested that N6-carbamoylthreonyladenosine levels, glycocholenate sulfate levels, C-glycosyltryptophan levels, ascorbic acid 2-sulfate levels, 3-hydroxyphenylacetoylglutamine levels, methyl vanillate sulfate levels, leucine levels, caprylate (8:0) levels, caproate (6:0) levels significantly increased the risk of LAS, whereas quinate levels, glycocholenate sulfate levels, C-glycosyltryptophan levels, 3-hydroxyphenylacetoylglutamine levels, eicosenedioate (C20:1-DC) levels, N-succinyl-phenylalanine levels, glycerol levels, 1-palmitoyl-2-linoleoyl-gpc (16:0/18:2) levels, adenosine 5′-diphosphate (ADP) to N-acetylglucosamine to N-acetylgalactosamine ratio, isoleucine to phosphate ratio had a negative impact on LAS strength significantly decreased the risk of LAS. Previous studies showing that leucine (Branched-Chain Amino Acid) levels were positively correlated with IS risk (21–23). Leucine concentration, particularly in the atherothrombotic subtype, also maintains high plasma after stroke (24). Leucine in particular, a branch chain amino acid, is essential for glutamic acid production in the brain because it donates amino groups to the process (25). A study examined the association between serum glycolithocholate sulfate levels and risk of atrial fibrillation, which can lead to LAS, among 1,919 Black participants in the Atherosclerosis Risk in Communities cohort study (26). Antioxidant qualities of a caffeoylquinic acid derivative can reduce lipid peroxidation and antioxidant enzyme activity, hence preventing brain ischemia (27). Patients with severe and complete ischemia exhibited significantly higher levels of glycerol lactate compared to patients without symptomatic ischemia; however, the findings differ from our MR study (28, 29).

The results of the study suggested that glycosyl ceramide (d18:1/23:1, d17:1/24:1) levels significantly increased the risk of CS, whereas ceramide (d18:1/24:1) levels, N-oleoylserine levels, lithocholate sulfate (1) levels, glutamate to glutamine ratio had a negative impact on CS strength significantly decreased the risk of CS. Sphingolipids, such as ceramide and its derivatives, glucosyl ceramide and ceramide-1-phosphate, have shown promise in inducing plaque inflammation and vascular events like myocardial infarction and IS (30, 31). Glutamate and the glutamine-to-glutamate ratio are independently associated with coronary artery disease, which closely associated with CS, and its severity in Chinese patients undergoing CAG (32).

The results of the study suggested that 6-hydroxyindole sulfate levels, 3-methoxycatechol sulfate (2) levels, 4-acetamidobutanoate levels, sphingosine levels, phenylpyruvate to 4-hydroxyphenylpyruvate ratio, alanine to asparagine ratio significantly increased the risk of SVS, whereas 7-alpha-hydroxy-3-oxo-4-cholestenoate (7-hoca) levels, 4-vinylguaiacol sulfate levels, Trans 3,4-methyleneheptanoate levels, 1-(1-enyl-palmitoyl)-2-linoleoyl-GPC (p-16:0/18:2) levels, carotene diol (2) levels, stearoyl sphingomyelin (d18:1/18:0) levels had a negative impact on CS strength significantly decreased the risk of SVS. Carotene diols, as antioxidants, have been recognized for their potential to mitigate various redox-mediated injuries and counteract the senescent phenotype, which appears to provide a potential explanatory link to the phenomenon of IS (33). Asparagine and alanine were found to be positively correlated with the National Institutes of Health Stroke Scale score using high-performance liquid chromatography to analyze the levels of amino acids in serum samples obtained from both healthy donors and patients diagnosed with IS (34).

The identification of particular metabolites as potential biomarkers holds great promise for the diagnosis, prognosis, and monitoring of IS treatment due to the ongoing discovery of metabolites linked to stroke. Various subtypes of IS exhibit distinct risk factors, and corresponding treatment protocols have been formulated (35). However, research regarding their pathogenesis and alterations in metabolite levels is currently constrained. Our study aims to investigate the impact of blood metabolites and metabolite ratios on IS subtypes, and provide researchers with a framework to explore the relationship between blood metabolites and metabolite ratios and IS subtypes. Future investigations should delve into the mechanisms and assess whether metabolites could serve as diagnostic biomarkers. Moreover, exploring therapeutic strategies targeting metabolites may improving patient symptoms and prognosis.

However, our study had several limitations. Firstly, the results of the MR study included some blood metabolites and metabolite ratios are currently no published findings in the literature, these results may serve as predictive indicators. Secondly, we cannot totally exclude pleiotropy and confounding variables in study outcomes, even when sensitivity analyses and confounders-related IVs are employed for correction. Thirdly, the analysis focuses solely on the causal relationships between metabolites and IS subtypes, without accounting for other relevant factors such as lifestyle or genetic predispositions. Future studies should integrate these additional factors to provide a more comprehensive understanding of the pathogenesis of IS subtypes. Finally, excluding IVW, many of the statistical findings lack significance, suggesting the necessity for additional data to facilitate more comprehensive research.

## 5 Conclusion

In conclusion, the present study assessed the possible causality of serum metabolites in the risk of IS subtypes. The discovery of significant causal links between 33 metabolites and 3 distinct IS subtypes. Metabolites show significant potential as circulating metabolic biomarkers and offer promise for clinical applications in the prevention and screening of IS subtypes. These discoveries notably advance our comprehension of the molecular processes specific to IS subtypes and create avenues for investigating targeted treatment approaches in the future.

## Data Availability

The original contributions presented in the study are included in the article/Supplementary material, further inquiries can be directed to the corresponding author.
