# Supplementary material for: Association of metabolites on ischemic stroke subtypes: a 2-sample Mendelian randomization study
Source: Front Neurol. 2024 Aug 29;15:1417357. doi: 10.3389/fneur.2024.1417357 (PMC11390583; doi:10.3389/fneur.2024.1417357)
Supplement: Supplementary file 2 [file Table_2.docx]

**STROBE-MR checklist of recommended items to address in reports of Mendelian randomization studies**^1^ ^2^

| **Item No.** | **Section** | **Checklist item** | **Relevant text from manuscript** |
| --- | --- | --- | --- |
| 1 | **TITLE and ABSTRACT** | Indicate Mendelian randomization (MR) as the study’s design in the title and/or the abstract if that is a main purpose of the study | Association of metabolites on ischemic stroke subtypes: A 2-sample Mendelian randomization study |
|  | **INTRODUCTION** |  |  |
| 2 | **Background** | Explain the scientific background and rationale for the reported study. What is the exposure? Is a potential causal relationship between exposure and outcome plausible? Justify why MR is a helpful method to address the study question | Metabolomics is increasingly being utilized in IS research to elucidate the intricate metabolic alterations that occur during ischemic stroke (IS). However, establishing causality in these associations remains unclear between metabolites and IS subtypes. |
| 3 | **Objectives** | State specific objectives clearly, including pre-specified causal hypotheses (if any). State that MR is a method that, under specific assumptions, intends to estimate causal effects | In this study, we employ Mendelian randomization (MR) to identify specific metabolites and investigate potential causal relationships between metabolites and IS subtypes. |
|  | **METHODS** |  |  |
| 4 | **Study design and data sources** | Present key elements of the study design early in the article. Consider including a table listing sources of data for all phases of the study. For each data source contributing to the analysis, describe the following: |  |
|  | a) | Setting: Describe the study design and the underlying population, if possible. Describe the setting, locations, and relevant dates, including periods of recruitment, exposure, follow-up, and data collection, when available. | MR analysis was conducted using genome-wide association study (GWAS) summary data. We obtained 1,091 blood metabolites and 309 metabolite ratios from the GWAS Catalog (GCST90199621-90201020), which gene sequencing data from 8,299 individuals from the Canadian Longitudinal Study. We obtained GWAS summary statistics for IS subtypes which include LAS, CES and SVS from the MEGASTROKE consortium that included 446,696 cases of European ancestry and 406,111 controls of European ancestry. T |
|  | b) | Participants: Give the eligibility criteria, and the sources and methods of selection of participants. Report the sample size, and whether any power or sample size calculations were carried out prior to the main analysis | We thank the IEU GWAS Catalog (https://www.ebi.ac.uk/ gwas/) initiative for developing and curating their data resources of plasma metabolome, accession numbers for European GWASs: GCST90199621-90201020. The MEGASTROKE project received funding from sources specified (http://www.megastroke.org/acknowledgments.html). |
|  | c) | Describe measurement, quality control and selection of genetic variants | Selection of IVs |
|  | d) | For each exposure, outcome, and other relevant variables, describe methods of assessment and diagnostic criteria for diseases | Selection of IVs |
|  | e) | Provide details of ethics committee approval and participant informed consent, if relevant | Ethics, approval, and consent to participate |
| 5 | **Assumptions** | Explicitly state the three core IV assumptions for the main analysis (relevance, independence and exclusion restriction) as well assumptions for any additional or sensitivity analysis | To investigate the causal effect of blood metabolites and metabolite ratios on IS across different subtypes, we obtain instrumental variables selection (IVs). Firstly, we selected SNPs with a correlation P < 1×10-5. Additionally, we applied a linkage disequilibrium (LD) threshold of R2 < 0.001 and a clumping distance of 10,000 kb by using “TwoSampleMR” packages. These stringent criteria ensured that the selected SNPs were independent and not in strong linkage disequilibrium with each other. By utilizing thresholds, we aimed to increase the number of eligible SNPs available for sensitivity analysis and to maximize the proportion of genetic variation that the genetic predictors could explain. After extracting the relevant information for each SNP, we calculated the proportion of interpreted variation (R2) and F statistics to quantify the strength of the instrumental variable. The F statistic is commonly employed to assess the effectiveness of instruments and is calculated using the formula F = R2 × (N− k -1)/ k (1 − R2), where R2 represents the proportion of variance explained by the instruments. It is calculated using the formula R2=2×MAF×(1−MAF)×β2, N represents the sample size and k denotes the number of selected IVs. In this study, we set a standard cutoff value of F statistic > 10 to mitigate the potential for weak instrument bias.  We employed different MR methods to assess the possible causal link between blood metabolites, metabolite ratios, and IS subtypes. We will further validate the results using four more MR approaches if the inverse variance weighted (IVW) method establishes a significant causal association (P < 0.01): Weighted median, basic mode, weighted mode, and MR-Egger13. These supplementary MR methods improve the consistency and robustness of our results. For identifying and addressing any biases brought about by pleiotropy, the MR-Egger approach is especially helpful. The weighted median approach provides a more reliable estimate when more than 50% of the IVs are invalid14. The estimates derived from various instrumental variables can be combined by employing either the simple or weighted mode methods as alternatives. By employing these multiple MR methods, we aim to obtain a comprehensive understanding of the potential causal connection between blood metabolites, metabolite ratios, and IS subtypes. Finally, odds ratios (OR) and 95% confidence intervals (CI) were used to present the results of causal connections.  Heterogeneity across estimates of genetic instruments may be assessed using funnel plots and Cochran's Q statistic, with a significant P-value threshold of 0.0510. Furthermore, we utilized the MR-Egger intercept test, employing a significant P-value cutoff of 0.05, to detect the presence of horizontal pleiotropy15. The results are visually presented using scatter plots16. To test the reliability of our conclusions, we implemented leave-one-out analyses, repeatedly performing the IVW analysis while excluding one exposure-related SNP at a time. This iterative approach enabled us to assess the robustness of our results by examining the individual SNP contributions to the causal connection between blood metabolites, metabolite ratios, and IS subtypes. By employing these combined methodologies, we aimed to ensure the validity and reliability of our findings14,17. Furthermore, to strengthen the reliability of our MR studies, we conducted replicated analyses by removing relevant confounders from IVs. Specifically, we obtained the confounder-related SNPs from the PhenoScanner V2 database. This step allowed us to address potential confounding factors and enhance the reliability of our MR analyses |
| 6 | **Statistical methods: main analysis** | Describe statistical methods and statistics used |  |
|  | a) | Describe how quantitative variables were handled in the analyses (i.e., scale, units, model) | Statistical analysis |
|  | b) | Describe how genetic variants were handled in the analyses and, if applicable, how their weights were selected | To investigate the causal effect of blood metabolites and metabolite ratios on IS across different subtypes, we obtain instrumental variables selection (IVs). Firstly, we selected SNPs with a correlation P < 1×10-5. Additionally, we applied a linkage disequilibrium (LD) threshold of R2 < 0.001 and a clumping distance of 10,000 kb by using “TwoSampleMR” packages. These stringent criteria ensured that the selected SNPs were independent and not in strong linkage disequilibrium with each other. By utilizing thresholds, we aimed to increase the number of eligible SNPs available for sensitivity analysis and to maximize the proportion of genetic variation that the genetic predictors could explain. After extracting the relevant information for each SNP, we calculated the proportion of interpreted variation (R2) and F statistics to quantify the strength of the instrumental variable. The F statistic is commonly employed to assess the effectiveness of instruments and is calculated using the formula F = R2 × (N− k -1)/ k (1 − R2), where R2 represents the proportion of variance explained by the instruments. It is calculated using the formula R2=2×MAF×(1−MAF)×β2, N represents the sample size and k denotes the number of selected IVs. In this study, we set a standard cutoff value of F statistic > 10 to mitigate the potential for weak instrument bias. |
|  | c) | Describe the MR estimator (e.g. two-stage least squares, Wald ratio) and related statistics. Detail the included covariates and, in case of two-sample MR, whether the same covariate set was used for adjustment in the two samples | Statistical analysis |
|  | d) | Explain how missing data were addressed | Statistical analysis |
|  | e) | If applicable, indicate how multiple testing was addressed | Statistical analysis |
| 7 | **Assessment of assumptions** | Describe any methods or prior knowledge used to assess the assumptions or justify their validity | We employed different MR methods to assess the possible causal link between blood metabolites, metabolite ratios, and IS subtypes. We will further validate the results using four more MR approaches if the inverse variance weighted (IVW) method establishes a significant causal association (P < 0.01): Weighted median, basic mode, weighted mode, and MR-Egger13. These supplementary MR methods improve the consistency and robustness of our results. For identifying and addressing any biases brought about by pleiotropy, the MR-Egger approach is especially helpful. The weighted median approach provides a more reliable estimate when more than 50% of the IVs are invalid14. The estimates derived from various instrumental variables can be combined by employing either the simple or weighted mode methods as alternatives. By employing these multiple MR methods, we aim to obtain a comprehensive understanding of the potential causal connection between blood metabolites, metabolite ratios, and IS subtypes. Finally, odds ratios (OR) and 95% confidence intervals (CI) were used to present the results of causal connections. |
| 8 | **Sensitivity analyses and additional analyses** | Describe any sensitivity analyses or additional analyses performed (e.g. comparison of effect estimates from different approaches, independent replication, bias analytic techniques, validation of instruments, simulations) | Heterogeneity across estimates of genetic instruments may be assessed using funnel plots and Cochran's Q statistic, with a significant P-value threshold of 0.0510. Furthermore, we utilized the MR-Egger intercept test, employing a significant P-value cutoff of 0.05, to detect the presence of horizontal pleiotropy15. The results are visually presented using scatter plots16. To test the reliability of our conclusions, we implemented leave-one-out analyses, repeatedly performing the IVW analysis while excluding one exposure-related SNP at a time. This iterative approach enabled us to assess the robustness of our results by examining the individual SNP contributions to the causal connection between blood metabolites, metabolite ratios, and IS subtypes. By employing these combined methodologies, we aimed to ensure the validity and reliability of our findings14,17. Furthermore, to strengthen the reliability of our MR studies, we conducted replicated analyses by removing relevant confounders from IVs. Specifically, we obtained the confounder-related SNPs from the PhenoScanner V2 database. This step allowed us to address potential confounding factors and enhance the reliability of our MR analyses |
| 9 | **Software and pre-registration** |  |  |
|  | a) | Name statistical software and package(s), including version and settings used | All analyses were carried out using the R 4.3.1 program. |
|  | b) | State whether the study protocol and details were pre-registered (as well as when and where) | None. |
|  | **RESULTS** |  |  |
| 10 | **Descriptive data** |  |  |
|  | a) | Report the numbers of individuals at each stage of included studies and reasons for exclusion. Consider use of a flow diagram | Figure1 |
|  | b) | Report summary statistics for phenotypic exposure(s), outcome(s), and other relevant variables (e.g. means, SDs, proportions) | None. |
|  | c) | If the data sources include meta-analyses of previous studies, provide the assessments of heterogeneity across these studies | None. |
|  | d) | For two-sample MR:  i.  Provide justification of the similarity of the genetic variant-exposure associations between the exposure and outcome samples  ii.  Provide information on the number of individuals who overlap between the exposure and outcome studies | None. |
| 11 | **Main results** |  |  |
|  | a) | Report the associations between genetic variant and exposure, and between genetic variant and outcome, preferably on an interpretable scale | We performed MR analysis to assess the causal association of 1,091 blood metabolites and 309 metabolite ratios with IS. The results assessed by the IVW-FE showed that N6-carbamoylthreonyladenosine levels (GCST90199762), N2,N2-dimethylguanosine levels (GCST90199764), 6-oxopiperidine-2-carboxylate levels (GCST90199949), palmitoyl dihydrosphingomyelin (d18:0/16:0) levels (GCST90200035), 1-stearoyl-2-docosahexaenoyl-gpc (18:0/22:6) levels (GCST90200046), ascorbic acid 2-sulfate levels (GCST90200094), methyl vanillate sulfate levels (GCST90200207), N-formylmethionine levels (GCST90200343), leucine levels (GCST90200389), caprylate (8:0) levels (GCST90200445), caproate (6:0) levels (GCST90200450), arachidonate (20:4n6) to paraxanthine ratio (GCST90200977) were associated with an increased risk for LAS, while quinate levels (GCST90199645), glycocholenate sulfate levels (GCST90199841), sphingomyelin (d18:1/20:2, d18:2/20:1, d16:1/22:2) levels (GCST90199995), C-glycosyltryptophan levels (GCST90200008), 1-oleoyl-2-linoleoyl-GPE (18:1/18:2) levels (GCST90200082), 3-hydroxyphenylacetoylglutamine levels (GCST90200162), eicosenedioate (C20:1-DC) levels (GCST90200245), N-succinyl-phenylalanine levels (GCST90200262), Glycerol levels (GCST90200325), 1-palmitoyl-2-linoleoyl-gpc (16:0/18:2) levels (GCST90200330), 1-palmitoyl-2-linoleoyl-GPI (16:0/18:2) levels (GCST90200332), X-17335 levels (GCST90200530), X-21733 levels (GCST90200587), glycochenodeoxycholate glucuronide (1) levels (GCST90200693), adenosine 5'-diphosphate (ADP) to N-acetylglucosamine to N-acetylgalactosamine ratio (GCST90200733), isoleucine to phosphate ratio (GCST90200868) were associated with a decreased risk for LAS. Sphingomyelin (d17:1/16:0, d18:1/15:0, d16:1/17:0) levels (GCST90200017), glycosyl ceramide (d18:1/23:1, d17:1/24:1) levels (GCST90200119), X-24565 levels (GCST90200645), glutamine to asparagine ratio (GCST90200787), were associated with an increased risk for CS, while cysteine-glutathione disulfide levels (GCST90199784), 1,2-dilinoleoyl-GPE (18:2/18:2) levels (GCST90200068), ceramide (d18:1/24:1) levels (GCST90200098), N-oleoylserine levels (GCST90200099), perfluorooctanesulfonate (PFOS) levels (GCST90200100), lithocholate sulfate (1) levels (GCST90200231), plasma free asparagine levels (GCST90200452), X-17653 levels (GCST90200553), X-21383 levels (GCST90200591), X-23782 levels (GCST90200618), lycochenodeoxycholate glucuronide (1) levels (GCST90200693), glutamate to glutamine ratio (GCST90200776) were associated with a decreased risk for CS. 6-hydroxyindole sulfate levels (GCST90200002), 3-methoxycatechol sulfate (2) levels (GCST90200007), dibutyl sulfosuccinate levels (GCST90200256), 4-acetamidobutanoate levels (GCST90200311), sphingosine levels (GCST90200385), X-12822 levels (GCST90200507), X-13866 levels (GCST90200527), aspartate to mannose ratio (GCST90200882), phenylpyruvate to 4-hydroxyphenylpyruvate ratio (GCST90200886), alanine to asparagine ratio (GCST90200992) were associated with an increased risk for SVS, while 7-alpha-hydroxy-3-oxo-4-cholestenoate (7-hoca) levels (GCST90199804), 4-vinylguaiacol sulfate levels (GCST90199982), trans 3,4-methyleneheptanoate levels (GCST90200023), 1-(1-enyl-palmitoyl)-2-linoleoyl-GPC (p-16:0/18:2) levels (GCST90200060), carotene diol (2) levels (GCST90200140), docosahexaenoylcarnitine (C22:6) levels (GCST90200146), stearoyl sphingomyelin (d18:1/18:0) levels (GCST90200335), X-23659 levels (GCST90200616), were associated with a decreased risk for SVS (Figure 2).  In addition, four additional methods, MR-Egger, weighted median, simple mode, and weighted mode, were performed to assess the causal effect of these 1,091 blood metabolites and 309 on LAS, CES, and SVS (supplementary materials 2). |
|  | b) | Report MR estimates of the relationship between exposure and outcome, and the measures of uncertainty from the MR analysis, on an interpretable scale, such as odds ratio or relative risk per SD difference | supplementary materials 2 |
|  | c) | If relevant, consider translating estimates of relative risk into absolute risk for a meaningful time period | None. |
|  | d) | Consider plots to visualize results (e.g. forest plot, scatterplot of associations between genetic variants and outcome versus between genetic variants and exposure) | supplementary materials 2-4 |
| 12 | **Assessment of assumptions** |  |  |
|  | a) | Report the assessment of the validity of the assumptions | To investigate the causal relationship between immune cell types and eczema, a two-sample MR analysis was carried out, with the IVW approach serving as the primary analytical strategy and possible confounders and horizontal pleiotropy removed. A total of 48 immunophenotypes associated with eczema were found (P < 1×10-5) (Figure 2). |
|  | b) | Report any additional statistics (e.g., assessments of heterogeneity across genetic variants, such as *I^2^*, Q statistic or E-value) | To investigate the causal relationship between immune cell types and eczema, a two-sample MR analysis was carried out, with the IVW approach serving as the primary analytical strategy and possible confounders and horizontal pleiotropy removed. A total of 48 immunophenotypes associated with eczema were found (P < 1×10-5) (Figure 2). |
| 13 | **Sensitivity analyses and additional analyses** |  |  |
|  | a) | Report any sensitivity analyses to assess the robustness of the main results to violations of the assumptions | We performed MR analysis to assess the causal association of 1,091 blood metabolites and 309 metabolite ratios with IS. The results assessed by the IVW-FE showed that N6-carbamoylthreonyladenosine levels (GCST90199762), N2,N2-dimethylguanosine levels (GCST90199764), 6-oxopiperidine-2-carboxylate levels (GCST90199949), palmitoyl dihydrosphingomyelin (d18:0/16:0) levels (GCST90200035), 1-stearoyl-2-docosahexaenoyl-gpc (18:0/22:6) levels (GCST90200046), ascorbic acid 2-sulfate levels (GCST90200094), methyl vanillate sulfate levels (GCST90200207), N-formylmethionine levels (GCST90200343), leucine levels (GCST90200389), caprylate (8:0) levels (GCST90200445), caproate (6:0) levels (GCST90200450), arachidonate (20:4n6) to paraxanthine ratio (GCST90200977) were associated with an increased risk for LAS, while quinate levels (GCST90199645), glycocholenate sulfate levels (GCST90199841), sphingomyelin (d18:1/20:2, d18:2/20:1, d16:1/22:2) levels (GCST90199995), C-glycosyltryptophan levels (GCST90200008), 1-oleoyl-2-linoleoyl-GPE (18:1/18:2) levels (GCST90200082), 3-hydroxyphenylacetoylglutamine levels (GCST90200162), eicosenedioate (C20:1-DC) levels (GCST90200245), N-succinyl-phenylalanine levels (GCST90200262), Glycerol levels (GCST90200325), 1-palmitoyl-2-linoleoyl-gpc (16:0/18:2) levels (GCST90200330), 1-palmitoyl-2-linoleoyl-GPI (16:0/18:2) levels (GCST90200332), X-17335 levels (GCST90200530), X-21733 levels (GCST90200587), glycochenodeoxycholate glucuronide (1) levels (GCST90200693), adenosine 5'-diphosphate (ADP) to N-acetylglucosamine to N-acetylgalactosamine ratio (GCST90200733), isoleucine to phosphate ratio (GCST90200868) were associated with a decreased risk for LAS. Sphingomyelin (d17:1/16:0, d18:1/15:0, d16:1/17:0) levels (GCST90200017), glycosyl ceramide (d18:1/23:1, d17:1/24:1) levels (GCST90200119), X-24565 levels (GCST90200645), glutamine to asparagine ratio (GCST90200787), were associated with an increased risk for CS, while cysteine-glutathione disulfide levels (GCST90199784), 1,2-dilinoleoyl-GPE (18:2/18:2) levels (GCST90200068), ceramide (d18:1/24:1) levels (GCST90200098), N-oleoylserine levels (GCST90200099), perfluorooctanesulfonate (PFOS) levels (GCST90200100), lithocholate sulfate (1) levels (GCST90200231), plasma free asparagine levels (GCST90200452), X-17653 levels (GCST90200553), X-21383 levels (GCST90200591), X-23782 levels (GCST90200618), lycochenodeoxycholate glucuronide (1) levels (GCST90200693), glutamate to glutamine ratio (GCST90200776) were associated with a decreased risk for CS. 6-hydroxyindole sulfate levels (GCST90200002), 3-methoxycatechol sulfate (2) levels (GCST90200007), dibutyl sulfosuccinate levels (GCST90200256), 4-acetamidobutanoate levels (GCST90200311), sphingosine levels (GCST90200385), X-12822 levels (GCST90200507), X-13866 levels (GCST90200527), aspartate to mannose ratio (GCST90200882), phenylpyruvate to 4-hydroxyphenylpyruvate ratio (GCST90200886), alanine to asparagine ratio (GCST90200992) were associated with an increased risk for SVS, while 7-alpha-hydroxy-3-oxo-4-cholestenoate (7-hoca) levels (GCST90199804), 4-vinylguaiacol sulfate levels (GCST90199982), trans 3,4-methyleneheptanoate levels (GCST90200023), 1-(1-enyl-palmitoyl)-2-linoleoyl-GPC (p-16:0/18:2) levels (GCST90200060), carotene diol (2) levels (GCST90200140), docosahexaenoylcarnitine (C22:6) levels (GCST90200146), stearoyl sphingomyelin (d18:1/18:0) levels (GCST90200335), X-23659 levels (GCST90200616), were associated with a decreased risk for SVS (Figure 2).  In addition, four additional methods, MR-Egger, weighted median, simple mode, and weighted mode, were performed to assess the causal effect of these 1,091 blood metabolites and 309 on LAS, CES, and SVS (supplementary materials 2). |
|  | b) | Report results from other sensitivity analyses or additional analyses | Firstly, the results of Cochran’s Q test indicated the absence of heterogeneity. Subsequently, eicosenedioate (C20:1-DC) levels, ceramide (d18:1/24:1) levels, dibutyl sulfosuccinate levels, 3-methoxycatechol sulfate (2) levels showed horizontal pleiotropy by using the MR-Egger intercept test (P > 0.05) and MR-PRESSO global test (P > 0.05). Finally, the leave-one-out analysis (P > 0.05) proved the unreliability of the MR data in 6-oxopiperidine-2-carboxylate levels, sphingomyelin (d18:1/20:2, d18:2/20:1, d16:1/22:2) levels, 1-stearoyl-2-docosahexaenoyl-gpc (18:0/22:6) levels, 1-oleoyl-2-linoleoyl-GPE (18:1/18:2) levels, 1-palmitoyl-2-linoleoyl-GPI (16:0/18:2) levels, arachidonate (20:4n6) to paraxanthine ratio, cysteine-glutathione disulfide levels, sphingomyelin (d17:1/16:0, d18:1/15:0, d16:1/17:0) levels, plasma free asparagine levels, glycochenodeoxycholate glucuronide (1) levels, glutamine to asparagine ratio since excluding some IV did shift the results. Reorganization of the forest plot after the presentation of these metabolites (Figure 3). |
|  | c) | Report any assessment of direction of causal relationship (e.g., bidirectional MR) | The results indicated that the causal effects of blood metabolites and metabolite ratios, with the exception of N2,N2-dimethylguanosine levels, Palmitoyl dihydrosphingomyelin (d18:0/16:0) levels, N-formylmethionine levels, 1,2-dilinoleoyl-GPE (18:2/18:2) levels, perfluorooctanesulfonate (PFOS) levels, docosahexaenoylcarnitine (C22:6) levels, aspartate to mannose ratio, remained significant (Figure 4, supplementary materials 4)). This approach helps to isolate the effects of blood metabolites and metabolite ratios on IS subtypes by accounting for the potential influences of these confounding factors. |
|  | d) | When relevant, report and compare with estimates from non-MR analyses | None. |
|  | e) | Consider additional plots to visualize results (e.g., leave-one-out analyses) | supplementary |
|  | **DISCUSSION** |  |  |
| 14 | **Key results** | Summarize key results with reference to study objectives | In conclusion, the present study assessed the possible causality of serum metabolites in the risk of IS subtypes. The discovery of significant causal links between 33 metabolites and 3 distinct IS subtypes. Metabolites show significant potential as circulating metabolic biomarkers and offer promise for clinical applications in the prevention and screening of IS subtypes. These discoveries notably advance our comprehension of the molecular processes specific to IS subtypes and create avenues for investigating targeted treatment approaches in the future. |
| 15 | **Limitations** | Discuss limitations of the study, taking into account the validity of the IV assumptions, other sources of potential bias, and imprecision. Discuss both direction and magnitude of any potential bias and any efforts to address them | However, our study had several limitations. Firstly, the results of the MR study included some blood metabolites and metabolite ratios are currently no published findings in the literature, these results may serve as predictive indicators. Secondly, we can't totally exclude pleiotropy and confounding variables in study outcomes, even when sensitivity analyses and confounders-related IVs are employed for correction. Finally, excluding IVW, many of the statistical findings lack significance, suggesting the necessity for additional data to facilitate more comprehensive research. |
| 16 | **Interpretation** |  |  |
|  | a) | Meaning: Give a cautious overall interpretation of results in the context of their limitations and in comparison with other studies | The identification of particular metabolites as potential biomarkers holds great promise for the diagnosis, prognosis, and monitoring of IS treatment due to the ongoing discovery of metabolites linked to stroke. Various subtypes of IS exhibit distinct risk factors, and corresponding treatment protocols have been formulated33. However, research regarding their pathogenesis and alterations in metabolite levels is currently constrained. Our study aims to investigate the impact of blood metabolites and metabolite ratios on IS subtypes. and provide researchers with a framework to explore the relationship between blood metabolites and metabolite ratios and IS subtypes. Future investigations should delve into the mechanisms and assess whether metabolites could serve as diagnostic biomarkers. Moreover, exploring therapeutic strategies targeting metabolites may improving patient symptoms and prognosis. |
|  | b) | Mechanism: Discuss underlying biological mechanisms that could drive a potential causal relationship between the investigated exposure and the outcome, and whether the gene-environment equivalence assumption is reasonable. Use causal language carefully, clarifying that IV estimates may provide causal effects only under certain assumptions | The identification of particular metabolites as potential biomarkers holds great promise for the diagnosis, prognosis, and monitoring of IS treatment due to the ongoing discovery of metabolites linked to stroke. Various subtypes of IS exhibit distinct risk factors, and corresponding treatment protocols have been formulated33. However, research regarding their pathogenesis and alterations in metabolite levels is currently constrained. Our study aims to investigate the impact of blood metabolites and metabolite ratios on IS subtypes. and provide researchers with a framework to explore the relationship between blood metabolites and metabolite ratios and IS subtypes. Future investigations should delve into the mechanisms and assess whether metabolites could serve as diagnostic biomarkers. Moreover, exploring therapeutic strategies targeting metabolites may improving patient symptoms and prognosis.  However, our study had several limitations. Firstly, the results of the MR study included some blood metabolites and metabolite ratios are currently no published findings in the literature, these results may serve as predictive indicators. Secondly, we can't totally exclude pleiotropy and confounding variables in study outcomes, even when sensitivity analyses and confounders-related IVs are employed for correction. Finally, excluding IVW, many of the statistical findings lack significance, suggesting the necessity for additional data to facilitate more comprehensive research. |
|  | c) | Clinical relevance: Discuss whether the results have clinical or public policy relevance, and to what extent they inform effect sizes of possible interventions | In conclusion, the present study assessed the possible causality of serum metabolites in the risk of IS subtypes. The discovery of significant causal links between 33 metabolites and 3 distinct IS subtypes. Metabolites show significant potential as circulating metabolic biomarkers and offer promise for clinical applications in the prevention and screening of IS subtypes. These discoveries notably advance our comprehension of the molecular processes specific to IS subtypes and create avenues for investigating targeted treatment approaches in the future. |
| 17 | **Generalizability** | Discuss the generalizability of the study results (a) to other populations, (b) across other exposure periods/timings, and (c) across other levels of exposure | All data examined in this research is comprehensively presented in this published article and its supplementary materials. |
|  | **OTHER INFORMATION** |  |  |
| 18 | **Funding** | Describe sources of funding and the role of funders in the present study and, if applicable, sources of funding for the databases and original study or studies on which the present study is based | This work was financially supported by the State Administration of Traditional Chinese Medicine the fifth batch of National Training Program for Outstanding Clinical Talents in Traditional Chinese Medicine (National Letter of TCM Practitioners (2022) No. 1), the TCM Outstanding Talents Program of Liaoning Province (No.29 (2021), Liaoning Health Office), the Liaoning Provincial Department of Education Basic Project (Grant No. 2023C014). |
| 19 | **Data and data sharing** | Provide the data used to perform all analyses or report where and how the data can be accessed, and reference these sources in the article. Provide the statistical code needed to reproduce the results in the article, or report whether the code is publicly accessible and if so, where | We thank the IEU GWAS Catalog (https://www.ebi.ac.uk/ gwas/) initiative for developing and curating their data resources of plasma metabolome, accession numbers for European GWASs: GCST90199621-90201020. The MEGASTROKE project received funding from sources specified (http://www.megastroke.org/acknowledgments.html). |
| 20 | **Conflicts of Interest** | All authors should declare all potential conflicts of interest | The authors declare that there are no conflicts of interest regarding the publication of this paper. |

This checklist is copyrighted by the Equator Network under the Creative Commons Attribution 3.0 Unported (CC BY 3.0) license.

1. Skrivankova VW, Richmond RC, Woolf BAR, Yarmolinsky J, Davies NM, Swanson SA, et al. Strengthening the Reporting of Observational Studies in Epidemiology using Mendelian Randomization (STROBE-MR) Statement. JAMA. 2021;under review.

2. Skrivankova VW, Richmond RC, Woolf BAR, Davies NM, Swanson SA, VanderWeele TJ, et al. Strengthening the Reporting of Observational Studies in Epidemiology using Mendelian Randomisation (STROBE-MR): Explanation and Elaboration. BMJ. 2021;375:n2233.
